# Disruption of airway lymphatics as a novel cause of impairment of airway clearance in severe asthma

**DOI:** 10.1186/2045-7022-3-S1-O5

**Published:** 2013-05-03

**Authors:** Masahito Ebina, Tohru Takahashi

**Affiliations:** 1 Department of Respiratory Medicine, Tohoku University Graduate School of Medicine, Japan; 2 Ishinomaki Red-Cross Hospital, Pathology Department, Japan

## Background

We previously reported the phenotypic distribution patterns of airway smooth muscles in severe asthmatics; with smooth muscle bundle thickening only in large airways and in whole airways [1] , by different mechanisms, hypertrophy and/or hyperplasia [2], which was reexamined recently to fit in 55 fatal asthmatics by James AL, *et al*. [3]. We also revealed that pulmonary lymphatics distributed in interlobular septa and subpleural lesions were destroyed by increased fibrosis in patients with idiopathic pulmonary fibrosis which would impair alveolar clearance [4] . In this context, we were interested in the alteration of airway lymphatics in severe asthmatics and hypothesized that increased smooth muscle bundles and fibrosis in the airway walls would disrupt airway lymphatics and impair airway clearance in these patients.

## Method

The autopsy lungs of severe asthmatics and controls were examined by immunohistochemistry to reveal the lymphatics and morphometry using an image analyzer system was applied to compare the distribution of airway lymphatics in the same level of airways among these asthmatics and controls. We also estimated the degree of airway smooth muscles and fibrosis around the airways which would interrupt or disrupt airway lymphatics.

## Results

The total area of airway lymphatics in each lung was found to be positively correlated with the airway radius. The distribution areas of lymphatics in larger airways of both types of asthmatics were significantly decreased than controls, and the severe asthmatics with increased muscle layers only in larger airways were found to have less lymphatics in these airways than the other group of severe asthmatics with increased smooth muscles in whole airways. The lymphatics around smaller airways were also reduced in both phenotypes of asthmatics without statistic difference. The airway lymphatics of these severe asthmatics were interrupted by both thickened muscle bundle layers and fibrotic tissues developed in the airway walls.

## Conclusion

These results indicate disrupted airway lymphatics as a novel cause of mucosal edema in severe asthmatics.
